# Editorial: LANDMARKS IN DEVELOPMENTAL BIOLOGY AND EVOLUTION

**DOI:** 10.2174/1389202911314060001

**Published:** 2013-09

**Authors:** A.J Durston

## II- Developmental Principles

While still at school, most of us are deeply impressed by the underlying principles that so beautifully explain why the chemical elements are ordered as they are in the periodic table, and may wonder, with the theoretician Brian Goodwin, *‘whether there might be equally powerful principles that account for the awe-inspiring diversity of body forms in the living realm’.* In fact the question of how an organism acquires its structure and form during embryogenesis is one of the most intriguing and challenging in Science. It is now becoming clear that there are indeed developmental principles. These define developmental constraints that limit the life forms that can evolve. These constraints operate above and beyond the constraints imposed by Darwinian Natural Selection. This second ‘mini hot topic’ presents three articles that report on three very different but equally extraordinary discoveries coming from radically different approaches to identifying developmental principles and elucidating developmental mechanisms. The first approach is genetics, as used so successfully in Drosophila. The genes show the way. The article by **Alain Prochiantz** in this volume deals with his serendipitous discovery and the exploration by himself and his collaborators of an amazing and totally unexpected principle concerning the transcription factors encoded by the famous homeobox family of genes. This is that these molecules directly mediate a very primitive signalling mechanism that copies information directly from one cell to another. Another approach is to investigate evolution. The invertebrate fossils found in the Burgess shale and other similar deposits blew away the classical view of evolution, revealed a Cambrian explosion, and gave us new and extremely surprising insights into what kinds of lifeforms previously existed and how the animal kingdom has developed, as explained in the article by **Graham Budd**, in this volume. A third approach is to use cell biology. The ways cells interact, move, differentiate and divide in an embryo should account for how the embryo is built. This approach has attracted eminent mathematicians and physicists to biology. It has been used by those who have chosen to work with the cellular slime mould Dictyostelium as a model system for embryonic development. This work has led to spectacular advances, as described in **my own** article which describes how investigating a very simple signalling mechanism has revealed mind blowing beauty and complexity in the development of this simple organism and has possibly indicated novel general principles for development.

